# Comparison of different microbiome analysis pipelines to validate their reproducibility of gastric mucosal microbiome composition

**DOI:** 10.1128/msystems.01358-24

**Published:** 2025-01-28

**Authors:** Konrad Lehr, Baptiste Oosterlinck, Chee Kin Then, Matthew R. Gemmell, Rolandas Gedgaudas, Jan Bornschein, Juozas Kupcinskas, Annemieke Smet, Georgina Hold, Alexander Link, on behalf of ENIGMA: European Network for the Investigation of Gastrointestinal Mucosal Alterations

**Affiliations:** 1 Department of Gastroenterology, Hepatology and Infectious Diseases, Otto-von-Guericke University Magdeburg, Magdeburg, Germany; 2 Laboratory of Experimental Medicine and Pediatrics, Faculty of Medicine and Health Sciences, University of Antwerp, Antwerp, Belgium; 3 MRC Oxford Institute for Radiation Oncology, Department of Oncology, University of Oxford, Oxford, United Kingdom; 4 Department of Radiation Oncology, Shuang Ho Hospital, Taipei Medical University, New Taipei City, Taiwan; 5 Centre for Genomic Research, University of Liverpool 4591 , Liverpool, United Kingdom; 6 Institute for Digestive Research, Lithuanian University of Health Sciences 230647 , Kaunas, Lithuania; 7 MRC Translational Immune Discovery Unit, MRC Weatherall Institute of Molecular Medicine, John Radcliffe Hospital, University of Oxford, Oxford, United Kingdom; 8 Microbiome Research Centre, University of New South Wales 7800 , Sydney, Australia; Katholieke Universiteit Leuven, Leuven, Belgium

**Keywords:** gastric cancer, *Helicobacter pylori*, 16S rRNA gene sequencing, DADA2, MOTHUR, QIIME2, pipeline comparison

## Abstract

Microbiome analysis has become a crucial tool for basic and translational research due to its potential for translation into clinical practice. However, there is ongoing controversy regarding the comparability of different bioinformatic analysis platforms and a lack of recognized standards, which might have an impact on the translational potential of results. This study investigates how the performance of different microbiome analysis platforms impacts the final results of mucosal microbiome signatures. Across five independent research groups, we compared three distinct and frequently used microbiome analysis bioinformatic packages (DADA2, MOTHUR, and QIIME2) on the same subset of fastQ files. The source data set encompassed 16S rRNA gene raw sequencing data (V1–V2) from gastric biopsy samples of clinically well-defined gastric cancer (GC) patients (*n* = 40; with and without *Helicobacter pylori* [*H. pylori*] infection) and controls (*n* = 39, with and without *H. pylori* infection). Independent of the applied protocol, *H. pylori* status, microbial diversity and relative bacterial abundance were reproducible across all platforms, although differences in performance were detected. Furthermore, alignment of the filtered sequences to the old and new taxonomic databases (i.e., Ribosomal Database Project, Greengenes, and SILVA) had only a limited impact on the taxonomic assignment and thus on global analytical outcomes. Taken together, our results clearly demonstrate that different microbiome analysis approaches from independent expert groups generate comparable results when applied to the same data set. This is crucial for interpreting respective studies and underscores the broader applicability of microbiome analysis in clinical research, provided that robust pipelines are utilized and thoroughly documented to ensure reproducibility.

**IMPORTANCE:**

Microbiome analysis is one of the most important tools for basic and translational research due to its potential for translation into clinical practice. However, there is an ongoing controversy about the comparability of different bioinformatic analysis platforms and a lack of recognized standards. In this study, we investigate how the performance of different microbiome analysis platforms affects the final results of mucosal microbiome signatures. Five independent research groups used three different and commonly used bioinformatics packages for microbiome analysis on the same data set and compared the results. This data set included microbiome sequencing data from gastric biopsy samples of GC patients. Regardless of the protocol used, *Helicobacter pylori* status, microbial diversity, and relative bacterial abundance were reproducible across all platforms. The results show that different microbiome analysis approaches provide comparable results. This is crucial for the interpretation of corresponding studies and underlines the broader applicability of microbiome analysis.

## INTRODUCTION

Elucidating the impact of the gut microbiota on human well-being has arguably become one of the most complex and intriguing topics in health sciences in the past decade (1–5). Bacteria present in the human body can exert either pathogenic or beneficial effects on the host, influencing progression, prognosis, and therapeutic outcomes of specific diseases (6–8). Due to the rapid development of next-generation sequencing approaches and their application within clinical research, our understanding of the human microbiome and its role in health and disease have grown exponentially, resulting in a myriad of sequencing data generated across the globe. Reproducible microbiome quantification can link microbial features to specific pathological conditions. A unique and well-understood relationship between a bacterial pathogen and a disease is *Helicobacter pylori* (*H. pylori*) and its role in gastric cancer (GC) development (9, 10). *H. pylori* was the first bacterium to be detected in the stomach. Shortly after its discovery, *H. pylori* was linked with gastric carcinogenesis, and a causal link was established between the bacteria and the disease. Today, the steps leading from chronic inflammation of the stomach to progression of mucosal changes toward GC are well understood. *H. pylori* promotes this process through a cascade of molecular and histopathological events. These include chronic gastritis, which may progress to atrophic gastritis, intestinal metaplasia and finally dysplasia and GC. Other bacteria become an increasing part of the gastric microbiota with the gastric mucosal environment changing during these stages and often *H. pylori* is undetectable at the latter stages (11–13). The model of *H. pylori* and GC microbiome exemplifies how collective understanding and technological advances in microbiome research are shaping diagnosis and treatment.

Recent microbiome studies rely heavily on advanced sequencing technologies, including bacterial 16S rRNA gene amplicon sequencing, which generates a large amount of information that requires downstream analysis using bioinformatic approaches. Examples of popular tools used for such analyses are MOTHUR (14), QIIME2 (15), or DADA2 (16). These analysis packages have been developed to merge forward and reverse sequence reads as well as filtering, de-noising, and trimming sequence reads. A crucial step remains the annotation of the output using a sequence reference database, to put the results in a biological context. There are also several choices for such reference databases including SILVA (17), Greengenes (18), and the Ribosomal Data Project (19). Unsurprisingly, it has been reported that the choice of analysis pipeline and taxonomic database can influence results (20, 21), but limited data exist to demonstrate how the different pipelines compare when analyzing the same data set. In particular, it remains to be determined whether different bioinformatic pipelines can lead to comparable conclusions about the microbial changes in the gastric mucosa.

In this study, we distributed the same set of fastQ files to five independent research groups. All partners in this network project utilized locally established 16S rRNA gene analysis pipelines and possessed extensive experience in 16S rRNA gene amplicon data analysis (22–26). The different partners of the project are all part of the ENIGMA study group and have independent expertise in performing microbiome analyses and are located in different institutions, including the Otto-von-Guericke University Magdeburg, the University of Antwerp, the University of Oxford, the University of Liverpool, the University of New South Wales, and the Lithuanian University of Health Sciences. The aim was to investigate whether results generated by different pipelines and using different taxonomic databases yielded consistent outcomes for gastric mucosa specimens. To do so, the clustering of the sequences, alpha and beta diversity, the different abundance profiles and enrichment in certain groups were compared. Additionally, we examined whether recent updates to the International Code of Nomenclature for Prokaryotes and subsequent changes in taxonomic classification had an impact on the results (27).

## MATERIALS AND METHODS

### Study cohort

**Fig 1:**
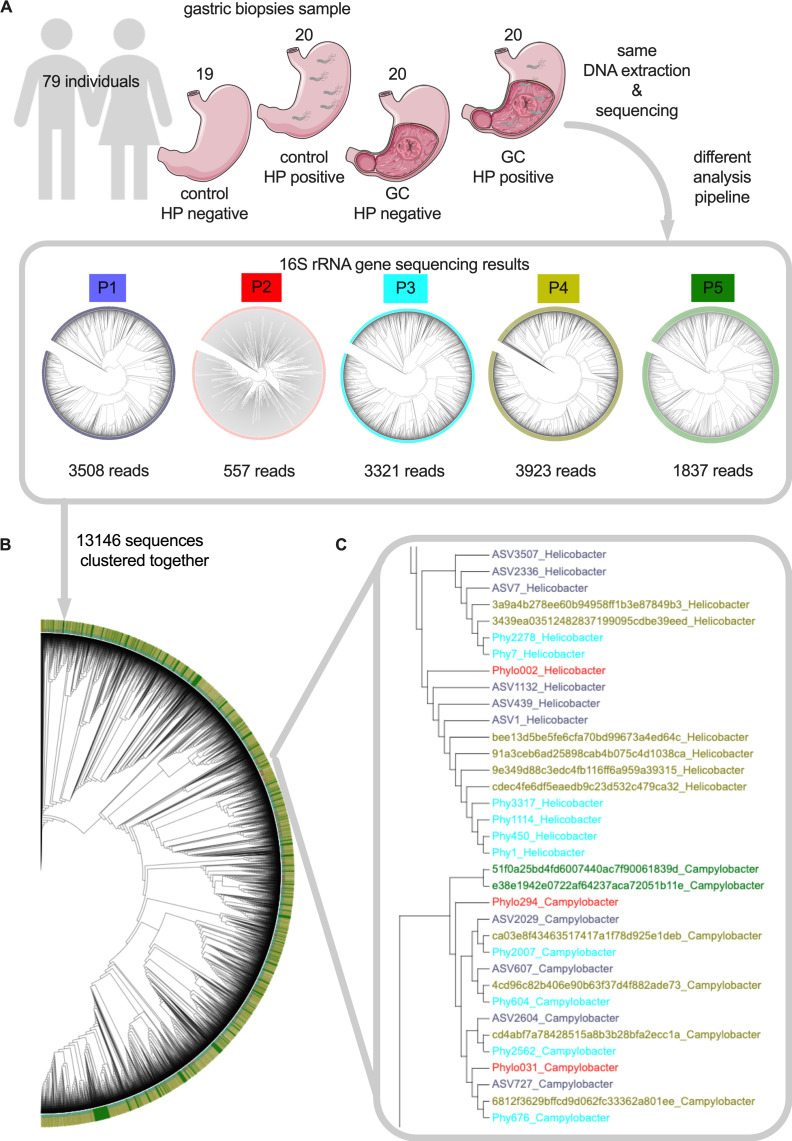
Sequence differentiation between analysis pipelines. Graphical abstract of the study (**A**). Phylogenetic tree of all V1–V2 16S rRNA gene sequences generated by all five project partners (**B**). Section of the entire tree (**C**). The sequences are colored for each project: P1 = gray, P2 = red, P3 = light blue, P4 = dark yellow, and P5 = green.

The study cohort consisted of 79 individuals (19 control HP-negative, 20 control HP-positive, 20 GC patients HP-negative, and 20 GC patients HP-positive); the full study design is shown in Fig. 1A and the detailed sampling procedure is explained elsewhere (28). Briefly, gastric biopsy specimens from malignant and healthy mucosa (i.e., at least 5 cm from the tumor tissue) were taken from GC patients using single-bite biopsy forceps. From the controls, healthy gastric mucosal biopsies were collected. All tissue samples were placed in sterile cryotubes, snap-frozen in liquid nitrogen, and stored at −80°C until further study. Subjects were recruited in 2012–2018 at the Department of Gastroenterology at the Lithuanian University of Health Sciences Kaunas. This study was approved by the local ethics committee of the Lithuanian University of Health Sciences (BE-2-10), and all participants gave written informed consent prior to the study.

### DNA and amplicon library preparation

Further processing of samples was performed at the Department of Gastroenterology, Hepatology, and Infectious Diseases at Otto-von-Guericke University (Magdeburg, Germany). Total DNA was extracted from gastric biopsy samples using an AllPrep DNA/RNA Mini Kit (Qiagen, Germany) according to the manufacturer’s recommendations. Amplicon libraries were constructed by amplifying the bacterial 16S rRNA gene V1–V2 region using the 27F and 338R (29) polymerase chain reaction primers. Sequencing was performed using a MiSeq (30) (2 × 250 bp; Illumina, Hayward, CA, USA).

### Bioinformatic analysis

The generated fastQ files were sent to the project partners, who each used their own pipeline for analysis. The different steps are provided in Table 1 and all generated count tables are added in Table S1. Briefly, the sequences were merged, filtered for different parameters (like number of *N*, expected errors), then trimmed to different lengths of 240–375 bp depending on the pipeline used and annotated with a taxonomic database. The annotation was carried out in each case with an older and a current version of the database (27). In addition, alpha-diversity (i.e., Simpson index, Shannon diversity, and species richness) and beta-diversity (i.e., Bray-Curtis distance metric) indices were calculated using the relative abundances for subsequent comparison. Bray-Curtis distance was used since it is the most sensitive distance measurement to observe differences between groups (31). Differential abundance analysis was done at the genus level using the linear discriminant analysis (LDA) effect size (LEfSe) algorithm implemented in the microbiomeMarker R-package v1.6.0 (32, 33).

**TABLE 1:** Comparison of analysis pipelines used by the different project partners (designated P1–P5)^*a*^

	P1	P2	P3	P4	P5
Platform	DADA2 (ee2) (16)	MOTHUR (14)	DADA2 (16)	QIIME2 (15)	QIIME2 (15)
Environment	Ubuntu 18.04.6 LTS CPU: Intel Core i7 R 3.6.1 with dada2 1.18	Ubuntu 20.04.6 LTS CPU: Intel(R) Xeon(R) W-2295 Mothur 1.48.0	Debian GNU/Linux 9.9 CPU: AMD EPYC 9754 128-Core Processor R 3.6.2 with dada2 1.12.1	Debian 11 bullseye CPU: AMD EPYC 7552 48-Core Processor R 4.3.2 with Qiime2-2020.2 and conda environment	macOS Monterey 12.7.3 CPU: Intel Core i5 Qiime2-2023.7 within a conda environment
Merged	mergePairs	make.contigs	mergePairs	Denoised “qiime DADA2 denoise-paired” (16) options: “--p-trunc-len-r 240,” “--p-trunc-len-f 200,” “--p-trim-left-r 17,” and “--p-trunc-q 2.”	VSEARCH plugin and join-pairs
Filter	– Denoised (learnErrors) – Chimera’s removal (removeBimeraDenovo) – Maximum *N* and expected errors (maxN = 0, maxEE = c(4:3))	– Filtering (screen.seqs) – Chimera’s removal (VSEARCH) – pre.cluster (fasta = current, count = current, diffs = 2)	– Denoised (learnErrors) – Chimera’s removal(removeBimeraDenovo) – Maximum *N* and expected errors (maxN = 0, maxEE = c (2, 2))		Deblur plugi (34) and denoise-16S method
Sequence length	240 bp (filterAndTrim and trimLeft)	Min: 275 bp Max: 375 bp	240 bp (filterAndTrim and trimLeft)	Min: 240 bp Max: 400 bp (TrimGalore option “-q 15”)	300 bp (cutadapt plugin and trim-paired)
Resampling	To 5,500 reads (Phyloseq R package) (35) excluding four samples not reaching required sequencing depth	Not applied	To 5,037 reads (Phyloseq R package) (35) excluding three samples not reaching required sequencing depth	To 5,584 reads (old taxonomy) and 6343 reads (new taxonomy).	Not applied
Clustering level	ASV	Phylotype (Genus)	ASV	ASV	ASV
Taxonomic annotation					
Database	Old and New: Ribosomal Data Project (19)	Old and New: SILVA (17)	Old and New: Ribosomal Data Project (19)	Old: Greengenes (18) New: Greengenes2 (36)	Old: Greengenes (18) New: SILVA (17)
NBC (37)	90%	90%	80%	Not applied	Not applied
Diversity	Vegan R package (38)	MOTHUR (14) (summary.single)	Vegan R package (38)	QIIME2 (15) (qiime diversity core-metrics-phylogenetic)	QIIME2 (15) (alpha-group-significance beta-group-significance)
Bray-Curtis matrix	Ecodist R package (39)	MOTHUR (14) (dist.shared)	Ecodist R package (39)		QIIME2 (15)

^*a*^ Our pipeline codes can be reviewed at https://doi.org/10.5281/zenodo.13911326. NBC, Naïve Bayesian classifier.

### Statistical analysis

All analyses were performed with abundances expressed as percentage. Phylogenetic trees were constructed with R (2022; R Foundation for Statistical Computing, Vienna, Austria) using the DECIPHER package (version 2.24.0) (40) and visualized in iTOL (41). Diversity comparison was visualized and tested for significance in Prism 9.2 (GraphPad Software, Boston, USA) using Kruskal-Wallis test. Pipeline-related differences were tested with the Friedman rank-sum test and the subsequent Kendall *W*’s effect size estimation and interpreted according to Cohen’s guidelines (<0.3 [small effect], 0.3–0.5 [moderate effect], and ≥0.5 [large effect]) (42). The applied significands level nomenclature for all tests is: ^ns^*P* > 0.05, * 0.049 > *P* > 0.01, ** 0.009 > *P* > 0.001, *** 0.0009 > *P* > 0.0001, and ***P < 0.0001. Stacked bar plots, Bray-Curtis heatmaps, and LEFse analysis were carried out and visualized using R with the packages ggplot2 (version 3.4.2) and microbiomeMarker (version 1.6.0), respectively.

To further clarify our workflow, we have completed the STORMS checklist (43) and published it at https://doi.org/10.5281/zenodo.13911326.

## RESULTS

### Different bioinformatics analysis pipelines produce comparable sequence results

The different bioinformatics platforms (Table 1) generated different numbers of unique sequences from the GC and control data sets, ranging from 557 (P2) to 3,923 (P4, Fig. 1A); the drastically reduced number of sequences in P2 can be explained by pipeline clustering at phylotype level. The 13,146 sequence reads generated across the partner data sets were then clustered in a single phylogenetic tree based on their similarity (Fig. 1B). A subset of frequently occurring sequences was selected to show that sequence similarity was not influenced by the bioinformatics platform used, as sequences from different projects cluster together (Fig. 1C). Otherwise, sequences from the individual projects should mainly cluster only with sequences from the same project. This was also evident from the fact that this subset of sequences was identified as *Helicobacter* and *Campylobacter* (depending on the branch, Fig. 1C). In summary, the sequence structure and similarity depend primarily on the bacterial 16S rRNA gene, while the bioinformatics platform used has only a minor influence.

### Loss of alpha-diversity in *H. pylori*-infected samples

**Fig 2:**
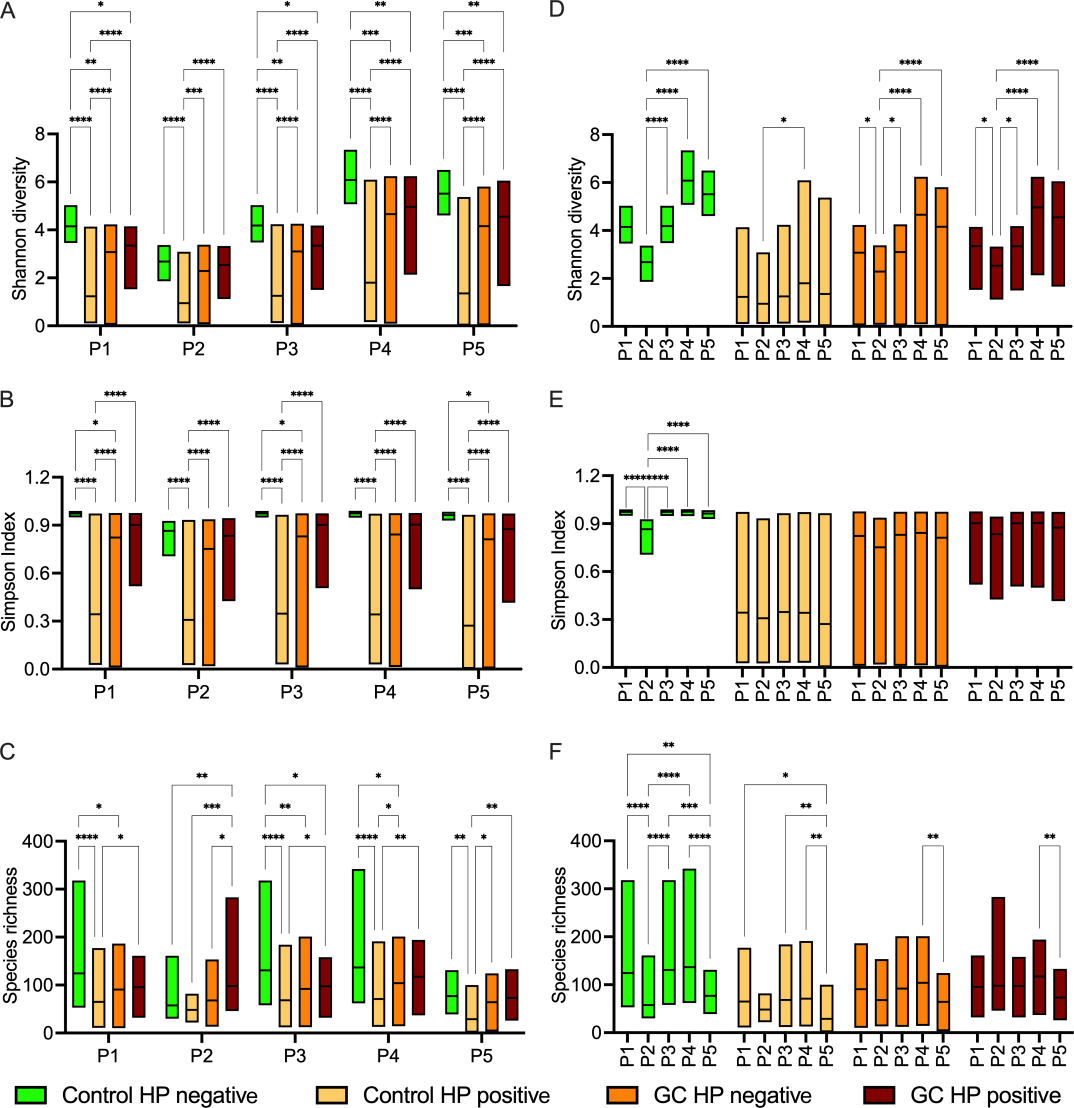
Comparison of diversity indices in gastric cancer patients and control subjects. Comparison of Shannon diversity (**A and D**), Simpson index (**B and E**), and species richness (**C and F**) based on the new taxonomy. (**A–C**) Grouping and statistical significance for comparison of sample groups and (**D–F**) for comparison of project partners.

Alpha-diversity (i.e., within samples) index values differed significantly between analysis pipelines (*P* < 1.19*10^−6^, Friedman rank-sum test) with moderate to large differences (*W* > 0.42; Kendall’s *W*). Regardless of the absolute differences, a significant loss of alpha-diversity in the *H. pylori*-positive control group (Fig. 2A through C) was found among all different analysis pipelines. Partners 4 and 5 (P4 and P5) reported significantly higher Shannon diversity (≈6) compared to P1, P3 (≈4), and P2 (≈3) in HP negative controls, as well as increased diversity in the GC groups (Fig. 2D) which may be linked to the QIIME2 platform. Such diversity differences between project partners were very limited for Simpson index (Fig. 2E). When assessing heterogeneity in species richness between data sets, P1, P3, and P4 found similar numbers of bacteria (≈130 in the *H. pylori*-negative controls, ≈70 in the *H. pylori*-positive controls, and ≈100 in the GC groups). P2 and P5 showed a lower number of bacteria in all groups, which can be attributed to different settings of the analysis pipeline, for example, sequence clustering at the phylotype level in P2, but the trends described above remain (Fig. 2C and F). Some pipelines produced different numbers of sequences depending on the taxonomy used, although this did not result in a significant difference in diversity measurements (Fig. S1). In summary, there were some differences between the projects in the calculation of the diversity indices, but these had no further influence on the overall results, showing the comparability of diversity data generated with different platforms.

### Gastric microbiome structure in all patient groups is independent of the bioinformatics platform and taxonomic database used

**Fig 3:**
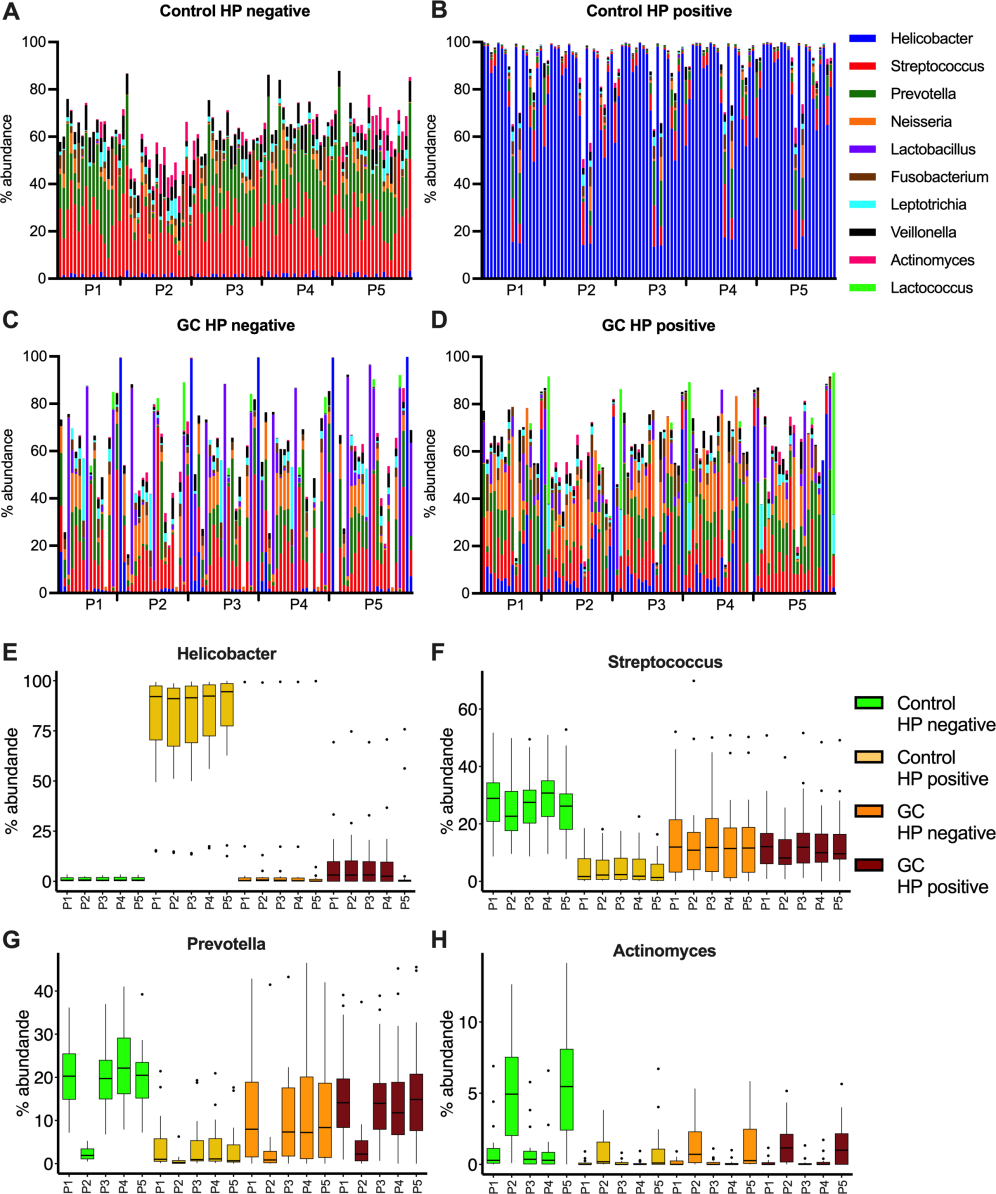
Bacterial assemblages in gastric cancer patients and controls. Relative bacterial abundance expressed as percentage for all samples at genus level, based on the new taxonomic classification. The 10 most abundant genera are selected for the different groups: Control HP negative (**A**), Control HP positive (**B**), GC HP negative (**C**), GC HP positive (**D**). The abundances of selected genera are displayed for the differences in groups and between projects (**E–H**).

Across all pipelines and databases used, 84 genera were reported with a relative abundance above 5% in at least one sample. Of these 84 genera, 10 were commonly reported across pipelines regardless of the database or the version used (i.e., *Actinomyces*, *Fusobacterium*, *Helicobacter*, *Lactobacillus*, *Lactococcus*, *Leptotrichia*, *Neisseria*, *Prevotella*, *Streptococcus*, and *Veillonella*; Fig. 3A through D according to the new taxonomy with old taxonomy in Fig. S2). The microbial community of the healthy stomach consisted of *Streptococcus*, *Prevotella*, and some less abundant bacteria such as *Neisseria* and *Veillonella* (Fig. 3A). The *H. pylori*-positive control cohort had a microbiota profile completely dominated by this bacterium, up to 82.5 ± 25.7 (in P5, Fig. 3B and E). GC patient samples had a higher heterogeneity in microbial structure regardless of *H. pylori* status, with a lower abundance of *Streptococcus*, *Prevotella*, or *Helicobacter*, but an increase in *Neisseria*, *Fusobacterium*, *Lactococcus*, or *Lactobacillus*. In addition, the increase in certain bacteria was heterogeneous in the GC cohort, making the microbiota more individualized for each patient (Fig. 3C and D).

Overall, comparable results were obtained with only minor differences identified between the different analysis approaches, for example, in genera like *Streptococcus* with no strong effects of the used pipeline (Fig. 3F). P2 and P5 found more *Actinomyces* and specially *Prevotella* was reduced in P2 when aligned with the new taxonomy database (Fig. 3G and H). The relative abundance of the 10 commonly reported genera was compared across pipelines (Table 2). In general, the effect of the bioinformatics pipeline on the relative abundance is small although this in part depends on the genus considered and can differ between patient populations. For example, when using the new taxonomy, *Actinomyces* relative abundance strongly differs between pipelines while this is not the case with the old taxonomy. When considering the effect size for *H. pylori* in the *H. pylori*-positive control group, the same large effect size was found when using both the new and old taxonomies.

**TABLE 2:** Effect size and significance of differences at genus level between pipelines^*a*^

Taxonomy	Genus	Controls				Gastric cancer			
		*H. pylori*−		*H. pylori*+		*H. pylori*−		*H. pylori*+	
New	*Actinomyces*	**0.66**	(****)	**0.62**	(****)	**0.60**	(****)	**0.70**	(****)
	*Fusobacterium*	0.06		0.28	(***)	0.27	(**)	0.28	(***)
	*Helicobacter*	0.07		**0.72**	(****)	0.09		0.24	(**)
	*Lactobacillus*	0.07		0.05		**0.31**	(***)	0.16	(*)
	*Lactococcus*	0.07		0.08		0.12		0.07	
	*Leptotrichia*	0.29	(***)	0.28	(***)	0.23	(**)	0.29	(***)
	*Neisseria*	0.16	(*)	0.22	(**)	0.26	(**)	0.26	(***)
	*Prevotella*	**0.72**	(****)	**0.69**	(****)	**0.46**	(****)	**0.54**	(****)
	*Streptococcus*	**0.46**	(****)	**0.44**	(****)	0.07		0.23	(**)
	*Veillonella*	**0.31**	(***)	0.21	(**)	0.27	(**)	0.22	(**)
Old	*Actinomyces*	0.17	(*)	0.20	(**)	0.26	(**)	**0.53**	(****)
	*Fusobacterium*	0.02		0.25	(***)	**0.32**	(***)	0.22	(**)
	*Helicobacter*	0.05		**0.72**	(****)	0.07		0.21	(**)
	*Lactobacillus*	0.06		0.05		0.18	(*)	0.21	(**)
	*Lactococcus*	0.07		0.05		0.09		0.06	
	*Leptotrichia*	**0.33**	(****)	**0.36**	(****)	**0.30**	(***)	**0.32**	(****)
	*Neisseria*	0.09		0.20	(**)	0.09		0.12	
	*Prevotella*	**0.68**	(****)	**0.62**	(****)	**0.37**	(****)	**0.59**	(****)
	*Streptococcus*	0.28	(***)	**0.46**	(****)	0.08		0.27	(***)
	*Veillonella*	**0.43**	(****)	0.30	(****)	**0.31**	(***)	0.27	(***)

^*a*^ For the 10 commonly reported genera, differences in relative abundance between bioinformatics pipelines were tested using the Friedman test. *P* values were corrected using Benjamini-Hochberg’s method (0.049 > *P > 0.01; 0.009 > **P > 0.001; 0.0009 > ***P > 0.0001; ****P < 0.0001), and effect size was calculated using Kendall *W*’s method (large values in bold font and underscored, medium values only in bold font, and small values in normal font).

The differences between the new and old taxonomies were limited, the percentage of sequences with the same genus annotation in both taxonomies ranged from 68% (P4) to 86% P1 (Fig. S3). Only minor abundance differences could be found on the genus level (Fig. S2 and S4). In the old taxonomy, most of the more common genera (21 genera) are found in all projects, whereas in the new taxonomy a large number of genera were found in P4 that only occur in this project (Fig. S5). The difference in the number of reported genera is partly due to discrepancies in database annotations and partly due to the analysis pipeline used.

**Fig 4:**
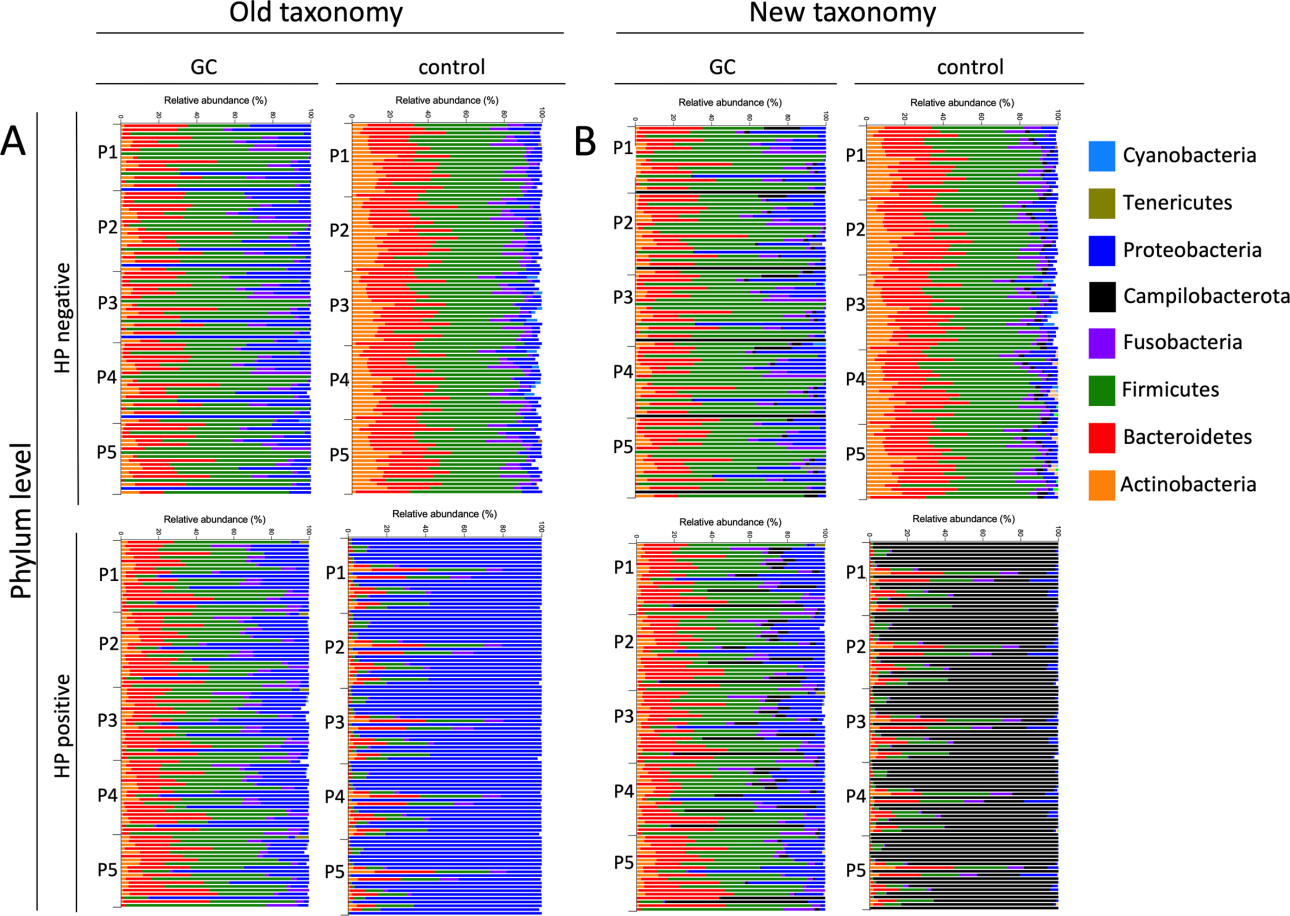
Taxonomical classification differs on phylum level. Relative bacterial abundance expressed as percentage for all samples at phylum level, based on the old (A) and new (B) taxonomic classification.

The results obtained at the phylum level unveiled less heterogeneity due to the higher taxonomic rank. Only the difference in taxonomy was more pronounced for the phylum Proteobacteria, as it initially included *H. pylori* based on the old taxonomy, but this bacterium is now assigned to the new phylum Campilobacterota (Fig. 4A and B).

### Comparable beta-diversity between patient samples among the bioinformatics platforms and taxonomies

**Fig 5:**
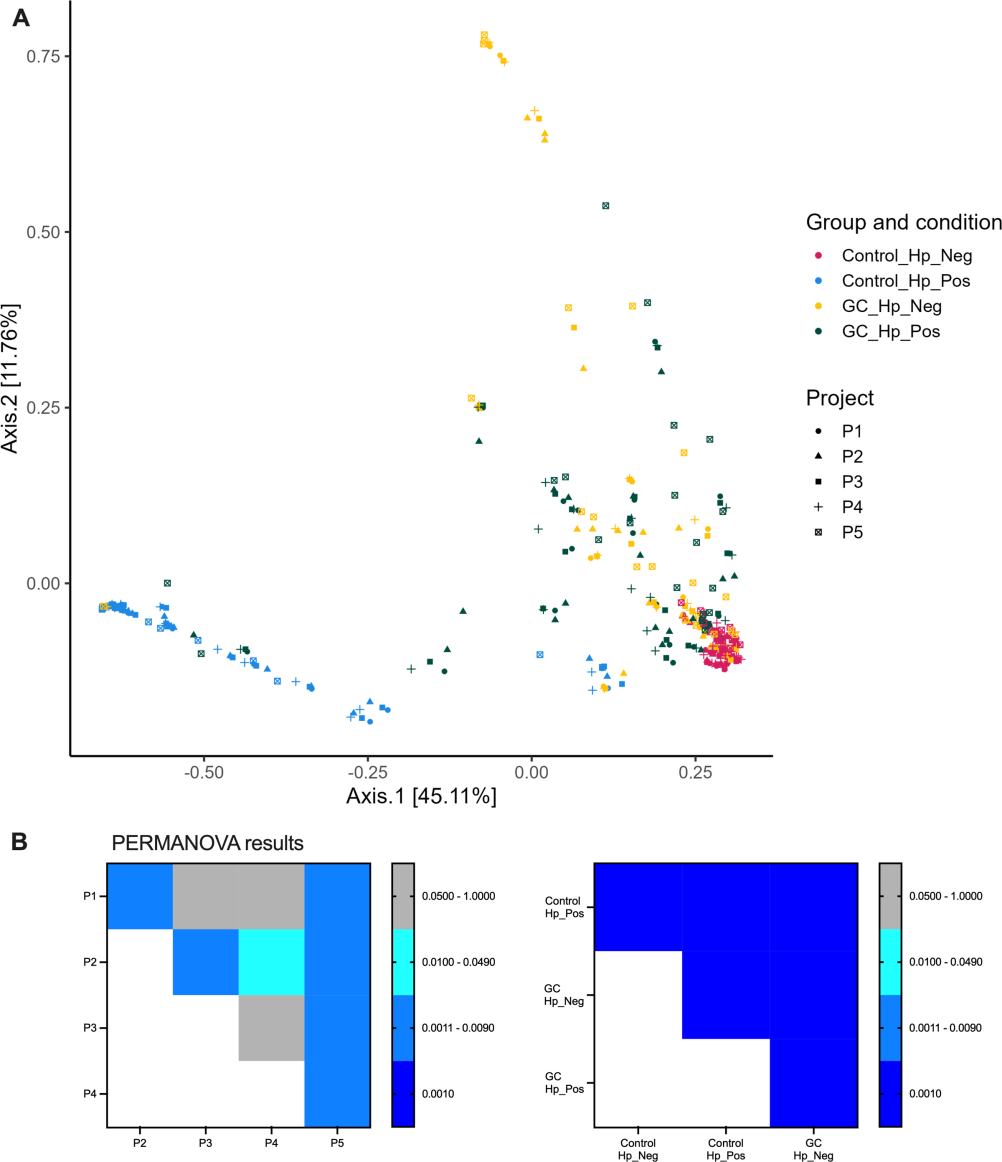
Beta-diversity between patient samples. PCO (**A**) and PERMANOVA (**B**) analyses based on Bray-Curtis distance measurement on the new taxonomy.

We next assessed the impact of the bioinformatic platform on beta-diversity analysis. Based on the bacterial structures described, Bray-Curtis distance analysis (i.e., beta- or between samples diversity measure) revealed strong similarity between samples of the *H. pylori*-positive control group, due to the high proportion of *H. pylori* in these samples. The microbial structure of the other sample groups was more heterogeneous and consequently these were more widely scattered, but the *H. pylori*-negative controls and both GC sample types were mostly distributed in opposite directions. The different taxonomies did not influence the overall Bray-Curtis distance analysis (Fig. 5A; Fig. S6). Furthermore, the PERMANOVA analysis showed some significant differences between the projects, especially with the P2 and P5 in the new taxonomy but not in the old taxonomy, and a consistent difference between the sample groups (Fig. 5B; Fig. S6).

On average, P1, P3, and P4 had a similar Bray-Curtis distance between all samples (67–68%), the overall distance of P2 and P5 was slightly increased (78% and 76%, respectively). These two pipelines detected a greater difference between the diversity of the control and GC samples. In addition, the Bray-Curtis distance between the same sample in different projects was always very low and smaller than to other samples of the same group (Fig. S7 and S8).

### Distinct microbial composition with different enrichment of genera in the GC and control cohorts

**Fig 6:**
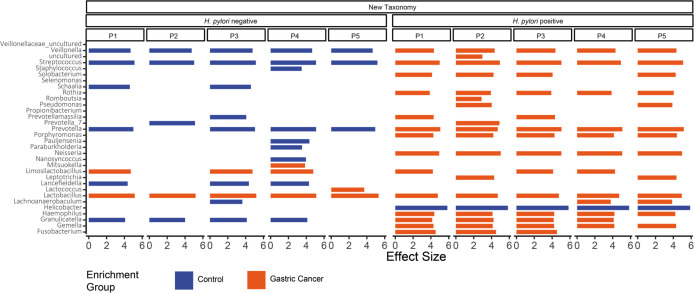
Bacterial enrichment in gastric cancer and control. LefSe analysis between control and GC sample groups based on the new taxonomic classification at genus level. All effect sizes shown reached significance level.

The LEfSe algorithm was performed to determine the bacterial taxa that were differentially enriched in GC patients compared to controls (Fig. 6; Fig. S9), with a total of 32 genera analyzed in the new and old taxonomies. Regardless of the taxonomy used, the enrichment in the control and GC groups differed largely by the *H. pylori* status of the sample. In the *H. pylori*-negative group, most genera were enriched in the controls and only a minority in the GC group, including *Mitsuokella* and *Lactobacillus*, but only the enrichment of *Lactobacillus* was reported for both taxonomies and for all projects. These genera also show enrichment for GC in the *H. pylori*-positive sample if detected. In the *H. pylori*-positive group, more genera showed enrichment, with all but *Helicobacter* was enriched in the GC group. The enrichment of *Helicobacter* in the control group is consistent with the previous results; logically, no enrichment of *Helicobacter* was detected for the *H. pylori*-negative samples. Twenty-five times the enrichment of a certain bacterium were detected in all pipelines, including *Helicobacter*, *Lactobacillus*, *Veillonella*, *Streptococcus*, and *Prevotella*, which are among the most abundant genera in the stomach. Even if the enrichment was not found in all projects, it was always found to occur in the same group, so that the overall results between the projects are comparable. The taxonomy used also had only a limited influence, but in some cases, enrichment was only detectable for one taxonomy (e.g., *Selenomonas* and *Pauliensenia*; Fig. S9).

## DISCUSSION

Comprehensive analysis of the microbial composition in the gastrointestinal tract is crucial for establishing associations between specific taxa and healthy and disease states (44). Amplicon sequencing of the 16S rRNA gene is currently the most widely used technology to unravel the microbial communities present in the human body (44). Microbial identification can be influenced by several factors, including the choice of analytical pipeline as this requires specialized technical and bioinformatics expertise, making comparison across studies challenging (45). Although several studies have evaluated the impact of different bioinformatics pipelines on microbial composition using simulated data sets or mock communities (46, 47), comparative approaches on real 16S rRNA gene data sets are currently limited and only based on the analysis of the fecal microbiome (21, 48, 49). Studies evaluating the impact of different bioinformatics pipelines on the gastric mucosal microbiome composition and the taxonomy analysis are still lacking. Therefore, in our study, we analyzed a subset of FastQ files, encompassing 16S rRNA gene raw sequencing data from biopsy samples of clinically well-defined GC patients and non-cancer controls, using different bioinformatics approaches performed by five independent and experienced groups. The sequences were clustered for similarity and analyzed for their bacterial diversity and the relative abundance of certain bacteria within samples and for their distance and the enrichment of bacteria between sample groups. The results from this study showed a clear dominance of *Helicobacter* in the *H. pylori*-positive controls, an enrichment of *Streptococcus*, *Prevotella* and to a lesser extent *Neisseria* or *Veillonella* in the *H. pylori*-negative control group and a greater heterogeneity in the microbiota composition with enrichment of *Lactococcus* among GC patients. All these findings are consistent with previous observations described in the literature (10, 22, 26, 28, 50–52).

Although there were some differences in the number of assigned reads, the number of distinct taxa, and the abundance of genera between the different analytical pipelines, an overall similar outcome for all applied tests, was noted. When comparing the reported reference sequences of the taxa of the different pipelines, significant differences were observed which are related to the clustering level (i.e., phylotype, OTU, or ASV). This is related to the differences in alpha-diversity. While sequence clustering at the phylotype level with MOTHUR (P2) resulted in a lower number of sequences and thus also lower diversity, clustering at the ASV level resulted in a higher number of sequences and thus also higher diversity in all pipelines. The pipelines with the DADA2 platform (P1 and P3) generated a comparable number of sequences, the difference in the QIIME2-based pipelines was greater (P4 and P5). The authors believe that this difference can be explained by the filtering method used in P5 (Denoise), which results in fewer reads and ultimately fewer species being found with this pipeline. Although the effects on the different indices were sometimes large, each pipeline allowed exactly the same conclusions to be drawn, as these are related to the relative differences in index values that were maintained between the pipelines.

When looking at the influence of analysis method on genus-level relative abundance, important differences become clear. Only 16.7% of the reported genera (with a minimum relative abundance >5% in at least one sample) were common between all pipelines. Interestingly, the chosen pipeline had little effect on the relative abundance of the most common genera (such as *Fusobacterium* or *Veillonella*), but our effect size analysis using the Kendall *W*’s method showed some clear differences in the abundance of the less common genera between pipelines. For example, the relative abundance of *Actinomyces* is significantly affected by the analysis pipeline, although each pipeline reported this genus as differentially abundant in the *H. pylori*-negative group. Caution should be exercised when comparing differentially abundant genera, as there are pipeline or database-specific genera for which the question remains whether there is a true biological difference.

Furthermore, for the first time, we have implemented the new taxonomic classification according to the International Code of Nomenclature of Prokaryotes (27). This question is relevant as the gastric mucosa may strongly be affected by the update. Overall, the update of the International Code of Nomenclature of Prokaryotes did not lead to fundamentally different results, but the interpretation and comparison of data can be difficult when taxa have been assigned to new families or phyla (27). Specifically, the key difference between the old and new taxonomies was the reclassification of *H. pylori* from *Proteobacteria* to the new phylum *Campylobacterota*. This underscores the importance of specifying the analytical methodology and metrics used in any clinical study.

Furthermore, the magnitude of the microbial differences identified in our study, is in line to what has been reported elsewhere with an overall similar biological outcome among the different used bioinformatics approaches as well (21, 48, 49). More specifically, comparative studies using distinct bioinformatics pipelines on fecal metagenomics data sets with different quality control options, clustering algorithms, and cutoff parameters, highlighted that DADA2 showed the best sensitivity, but lower specificity compared to QIIME2 (21). Furthermore, the integration of DADA2 in QIIME2 even has been shown to drastically improve the analyzed results of the raw sequencing data allowing the most accurate assignment of reads to be obtained and a better taxonomic resolution to be reached (45). MOTHUR also performed well at taxonomy level, although with lower specificity than the two other pipelines (21).

Besides the potential importance of bioinformatic pipelines, it is also important to consider the sample origin. Our analyses comprised gastric mucosa specimens with low to moderate microbial abundance, which may be of potential challenge compared to high microbial abundance specimens. The vast majority of previous studies focused on the analysis of fecal specimens or mock communities (20, 21, 53). Abellan-Schneyder et al. (20) compared the influence of different primer pairs and bioinformatic tools on microbiome analysis based on mock bacterial communities and fecal samples. Prodan et al. (21) tested different bioinformatics pipelines with different quality control options, clustering algorithms, and cutoff parameters on a mock community and a previously published fecal sample data set. Lopez-Garcia et al. (53) compared two commonly used pipelines for rumen microbiota composition analysis, suggesting the SILVA database as the preferred data set for classifying OTUs. Although comparable richness and diversity were provided by QIIME and MOTHUR, the greatest differences were found for the less abundant microorganisms which may be particularly relevant with mucosal specimens. The only study exploring caecal mucosal specimens evaluated different sequencing platforms was performed in chicken and may not be applied to humans in particular with an impact on the disease-related changes (49), which makes our work unique and translationally relevant.

Besides the choice of the bioinformatics pipeline for downstream analysis, other parameters can impact taxa identification and quantification. These include primer selection, to amplify a region within the 16S rRNA gene which has even a far greater impact on the microbiome results than when using different bioinformatics pipelines simultaneously (20, 44). Primers targeting the V3–V4 region of the 16S rRNA gene are currently the most widely used. However, primers targeting the V1–V2 region of the 16S rRNA gene should be the preferred choice when using DNA extracted from human biopsy specimens, as was the case in our study. In fact, these primers result in approximately 80% fewer reads aligning with the human genome, allowing for a more statistically significant analysis of the bacterial communities residing in tissue samples with 95% human DNA and 5% bacterial DNA. Furthermore, V1–V2 primers showed a superior performance in identifying more taxa and had a better resolution sensitivity for *Streptococcus* in the oral microbiome than V3–V4 primers (20, 44).

From a clinical perspective, the question remains how are the findings applicable for analysis in real-life settings of the human microbiome and how can data be compared for specific body sites/compartments? Considering the potential of aligning microbiome study findings and the application of re-evaluation of studies as the microbiome field advances, it is reasonable to assess whether different research groups come to comparable conclusions after systematic analysis of similar sample types—mucosal biopsies/swabs or fecal samples as examples. This knowledge does not however overcome upstream biases introduced through sample storage/handling and processing which must also be considered (54–56).

Here, we had a unique experiment with real human samples and were able to eliminate the biases due to sample storage, extraction, primer selection, and sequencing as all samples were handled in one place, allowing us to focus on the influence of the bioinformatics pipeline used. Overall, we acknowledge that the MOTHUR-based pipeline of P2 has the most differences compared to the other pipelines that gave comparable results within one platform and also between DADA2 and QIIME2. However, since each pipeline used reached the same conclusions within itself (e.g., Dominance of *Helicobacter*, loss of diversity, and negligible sample spacing in the *H. pylori*-positive group), we cannot commit to a preferred pipeline from a clinical perspective, nor can we recommend which pipeline to use. Rather, the results of our work show that the analyses of the luminal microbiome across different bioinformatics platforms demonstrate robust and reproducible results in the gastric microbiome model, supporting its reliability for real-world clinical research applications. The limited influence of taxonomic database choice further underscores the stability of microbiome analysis results, confirming its suitability for translational studies in patients with GC or inflammatory and preneoplastic conditions.

## Data Availability

The raw 16S rRNA gene sequencing data that support the findings of this study are available from the NCBI Sequence Read Archive (BioProject: PRJNA1096273).

## SUPPLEMENTAL MATERIAL

Supplemental Figures msystems.01358-24-s0001.pdf Figures S1 to S9.

Table S1 msystems.01358-24-s0002.xlsx All pipeline outputs.

ASM does not own the copyrights to Supplemental Material that may be linked to, or accessed through, an article. The authors have granted ASM a non-exclusive, world-wide license to publish the Supplemental Material files. Please contact the corresponding author directly for reuse.
