# Supplementary material for: Comparison of different microbiome analysis pipelines to validate their reproducibility of gastric mucosal microbiome composition
Source: mSystems. 2025 Jan 28;10(2):e01358-24. doi: 10.1128/msystems.01358-24 (PMC11834405; doi:10.1128/msystems.01358-24)
Supplement: Supplemental Figures — Figures S1 to S9. [file msystems.01358-24-s0001.pdf]

Comparison of different microbiome analysis pipelines to validate their reproducibility of gastric mucosal microbiome composition

Konrad Lehr[1]*, Baptiste Oosterlinck[2]*, Chee Kin Then[3,4]*, Matthew R. Gemmell[5]*, Rolandas Gedgaudas[6]*, Jan Bornschein[7], Juozas Kupcinskas[6], Annemieke Smet[2], Georgina Hold[8], Alexander Link[1]
on behalf of ENIGMA: European Network for the Investigation of Gastrointestinal Mucosal Alterations.

1 Department of Gastroenterology, Hepatology and Infectious Diseases
Otto-von-Guericke University Magdeburg, Germany
2 Laboratory of Experimental Medicine and Pediatrics,
Faculty of Medicine and Health Sciences, University of Antwerp, Belgium
3 MRC Oxford Institute for Radiation Oncology, Department of Oncology,
University of Oxford, United Kingdom
4 Department of Radiation Oncology, Shuang Ho Hospital,
Taipei Medical University, New Taipei City, Taiwan
5 Centre for Genomic Research, University of Liverpool, Liverpool, United Kingdom
6 Institute for Digestive Research,
Lithuanian University of Health Sciences, Kaunas, Lithuania
7 MRC Translational Immune Discovery Unit,
MRC Weatherall Institute of Molecular Medicine,
John Radcliffe Hospital, University of Oxford, United Kingdom
8 UNSW Microbiome Research Centre, University of New South Wales, Sydney,
Australia

*authors contributed equally

CORRESPONDING AUTHORS:
Konrad Lehr Email: konrad.lehr@med.ovgu.de
Alexander Link (MD, Ph.D.) Email: alexander.link@med.ovgu.de

Department of Gastroenterology, Hepatology and Infectious Diseases
Medical Faculty, Otto-von-Guericke University Magdeburg
Leipziger Straße 44
39120 Magdeburg
Tel.: +49 391 67 13100

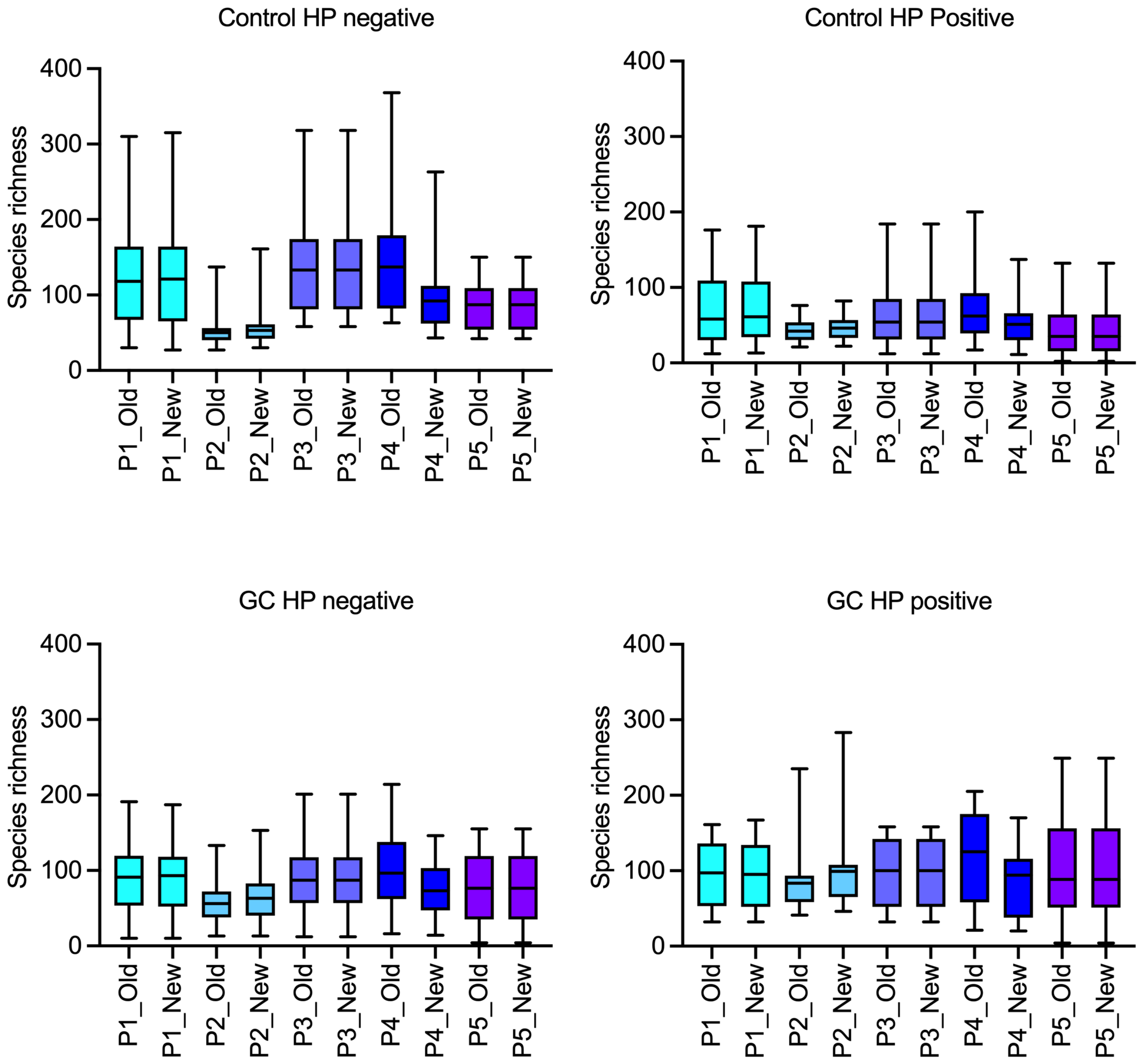

Supplementary Figure 1: Comparison of the species richness based on the old and new taxonomy.

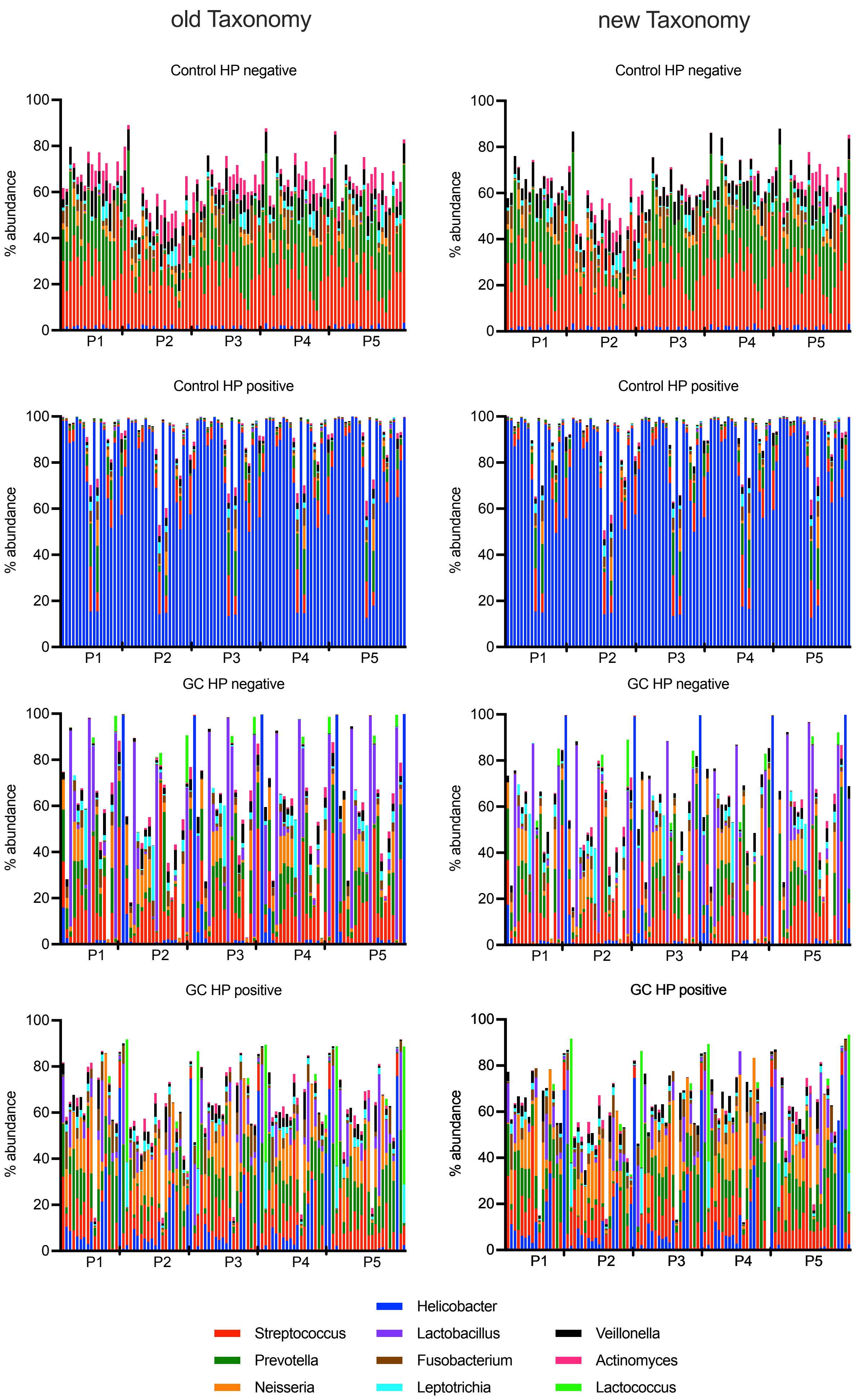

Supplementary Figure 2: Bacterial assemblages in gastric cancer patients and controls. Relative bacterial abundance for all samples at genus level, based on the old and new taxonomic classification. The 10 most abundant genera were selected.

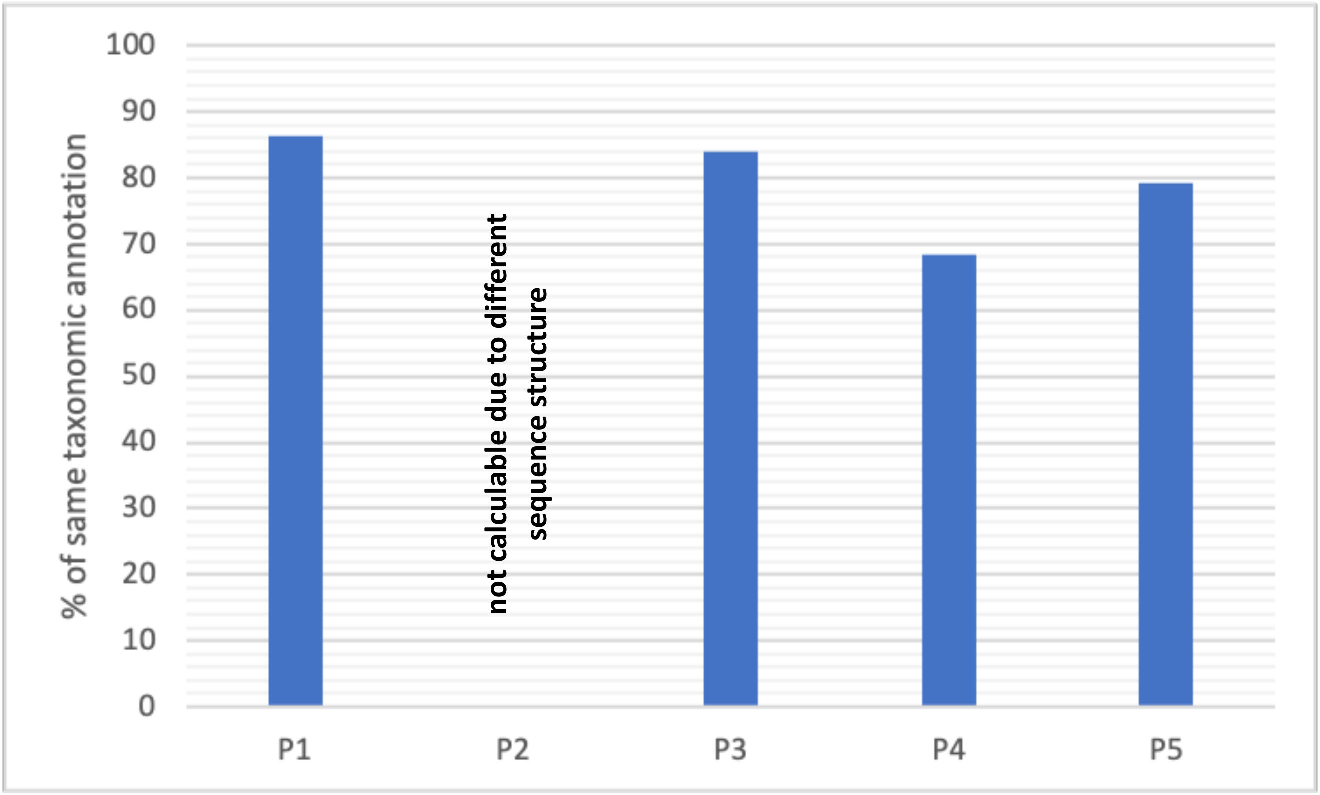

Supplementary Figure 3: Percentage of the same genus annotated to a sequence according to the old and the new taxonomy.

Old Taxonomy

New Taxonomy

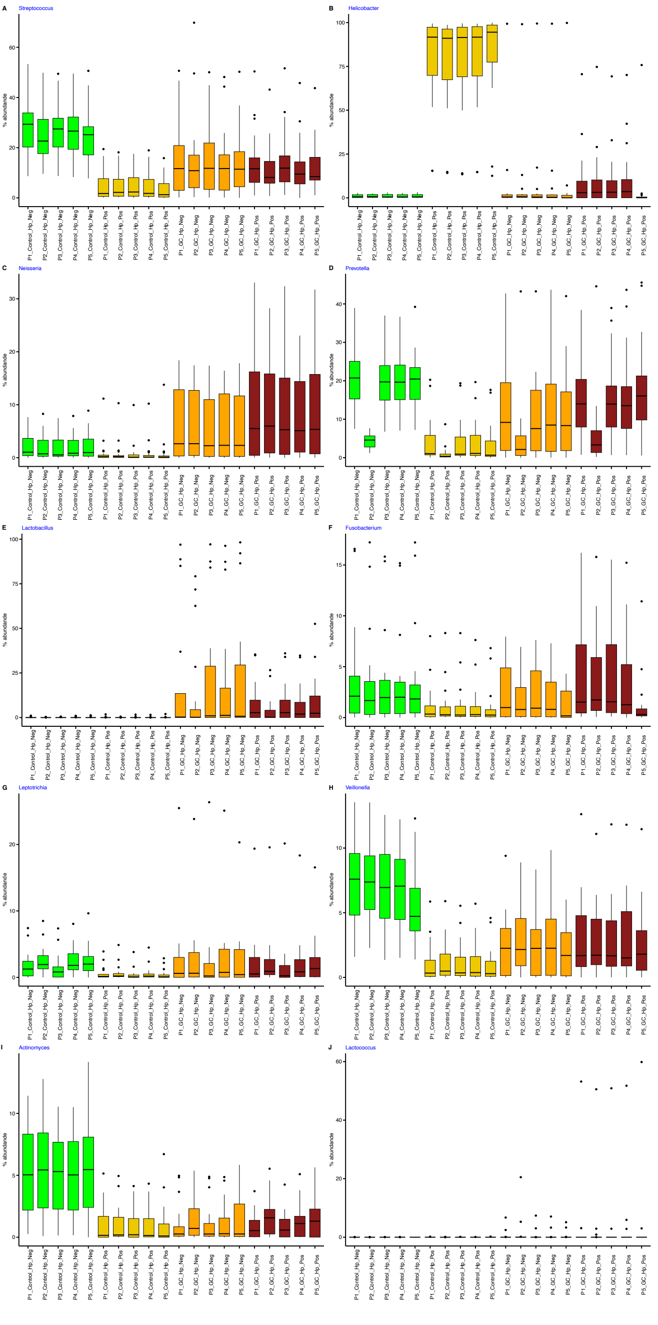

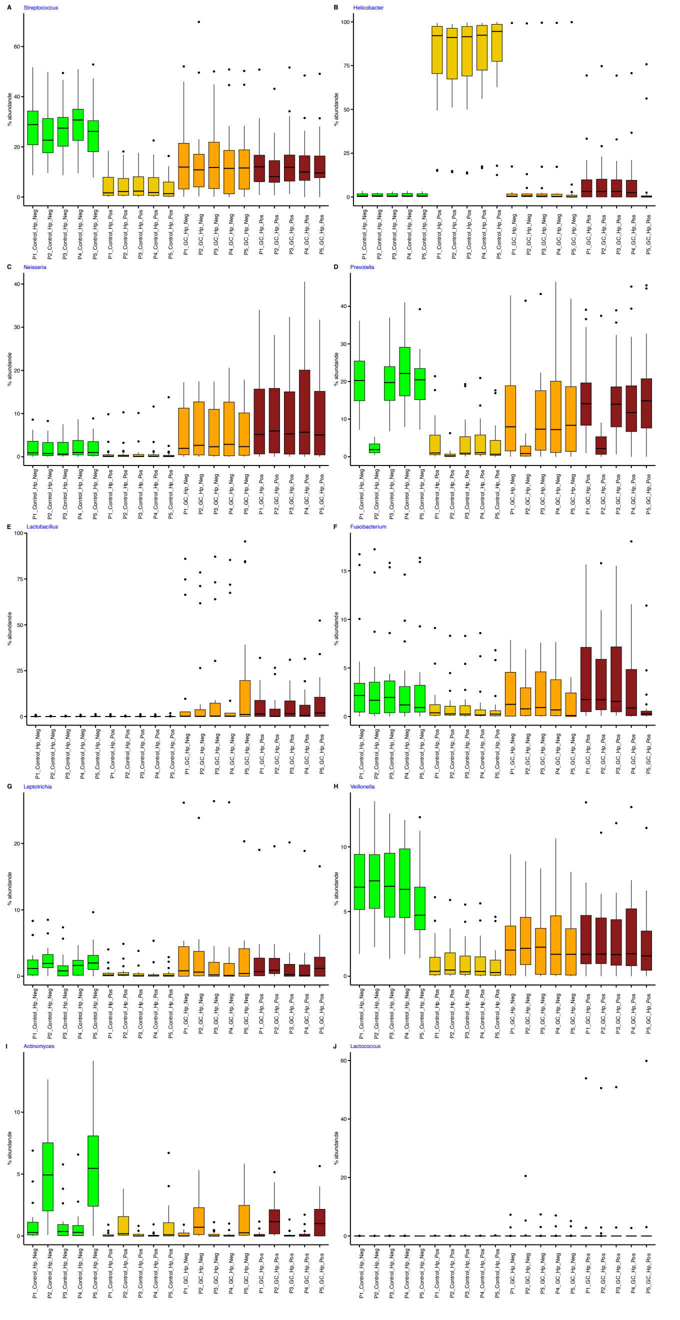

Supplementary Figure 4: Abundance of the 10 most abundant genera for each group and project. The groups are displayed in different colours (Control HP negative = green, Control HP positive = yellow, GC HP negative = orange, GC HP positive = dark red).

Old taxonomy

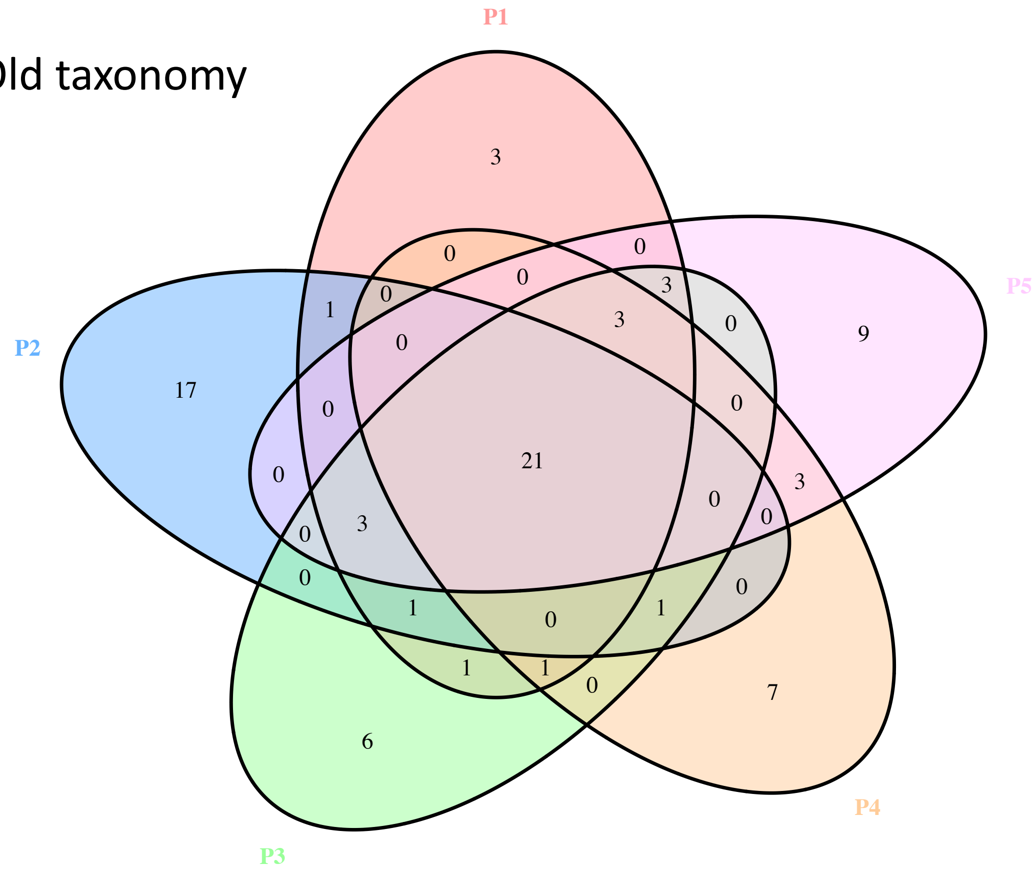

New taxonomy

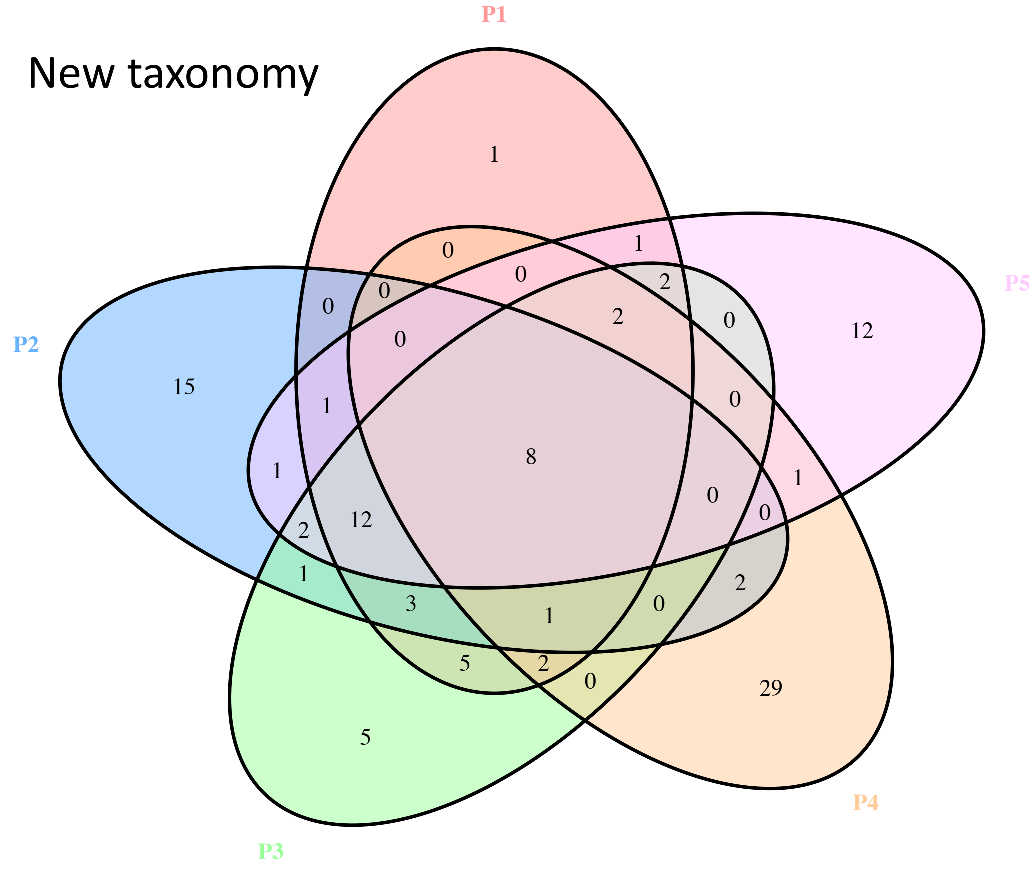

Supplementary Figure 5: Venn diagram of shared genera between projects with an abundance higher than 5% according to the old and the new taxonomy.

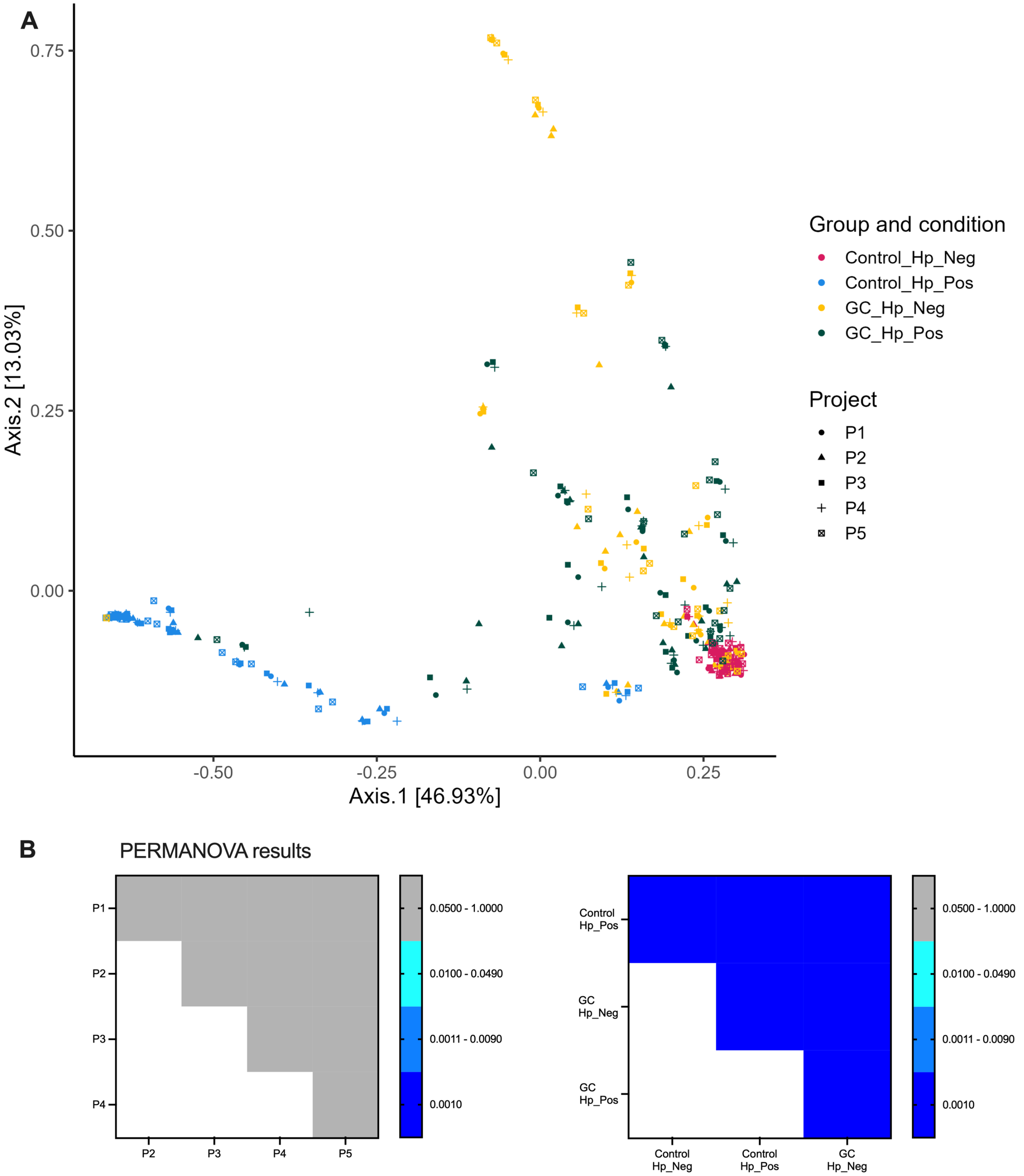

Supplementary Figure6: PCO and PERMANOVA analysis based on Bray-Curtis distance measurement on the old taxonomy.

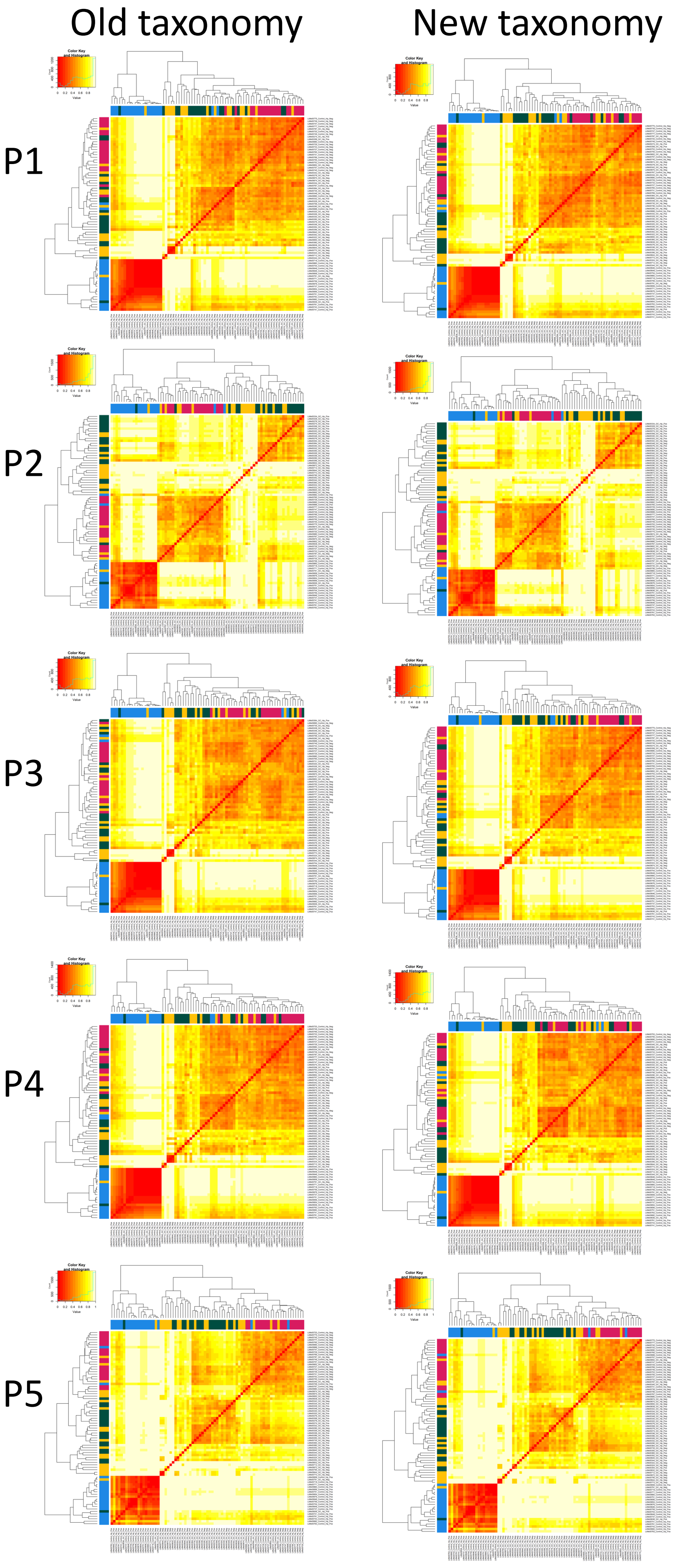

Supplementary Figure 7: Similarity analysis between different specimen types. Heatmaps showing the Bray-Curtis distance between all samples at genus level, based on the old and new taxonomic classification. Sample groups are shown in blue (control-HP positive), red (control-HP negative), dark green (GC-HP positive) and yellow (GC-HP negative).

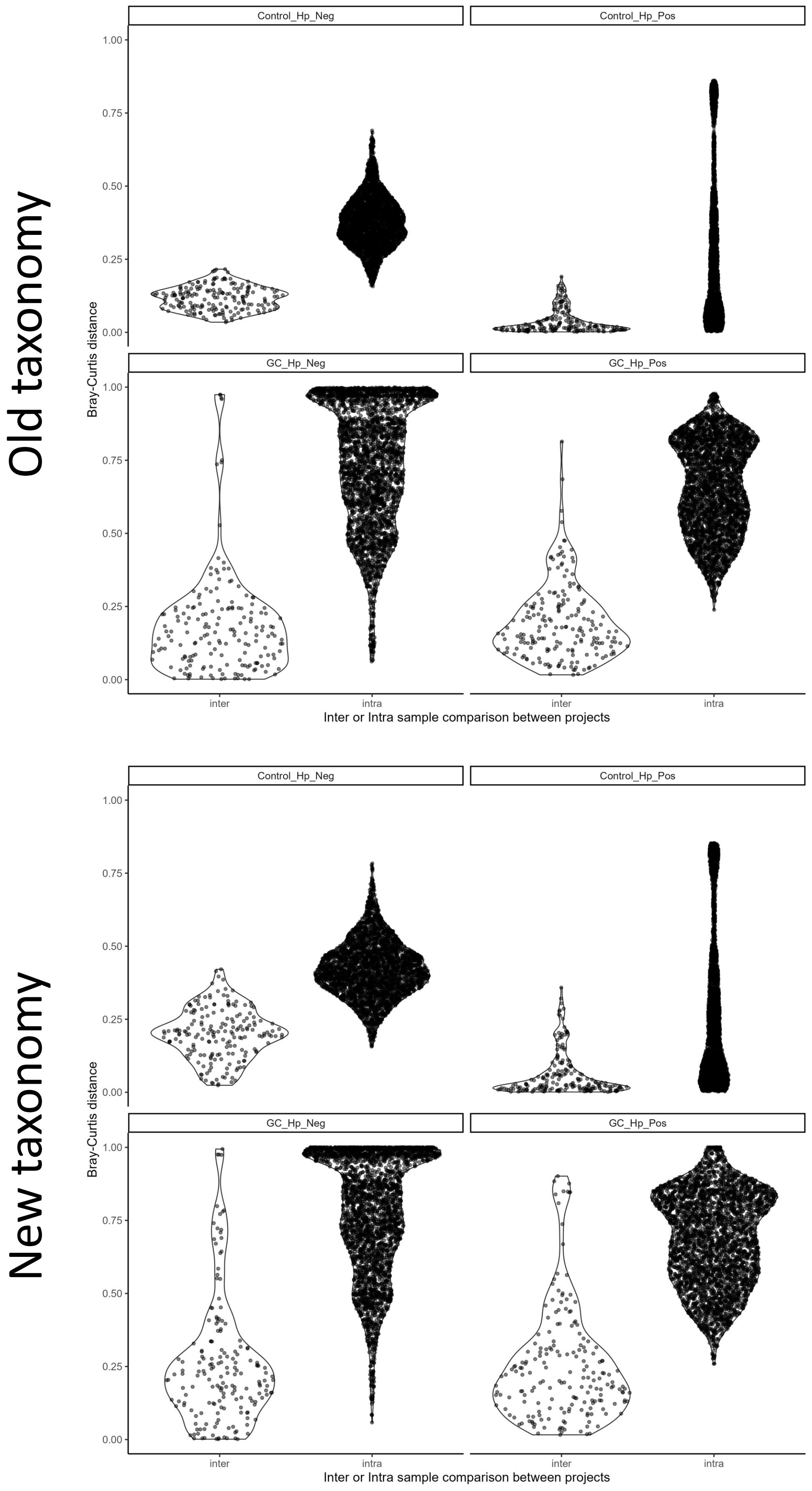

Supplementary Figure 8: Violin plots of Bray-Curtis distance between the same sample in different projects (inter) and between other sample of the same group in different projects (intra), based on the old and new taxonomic classification.

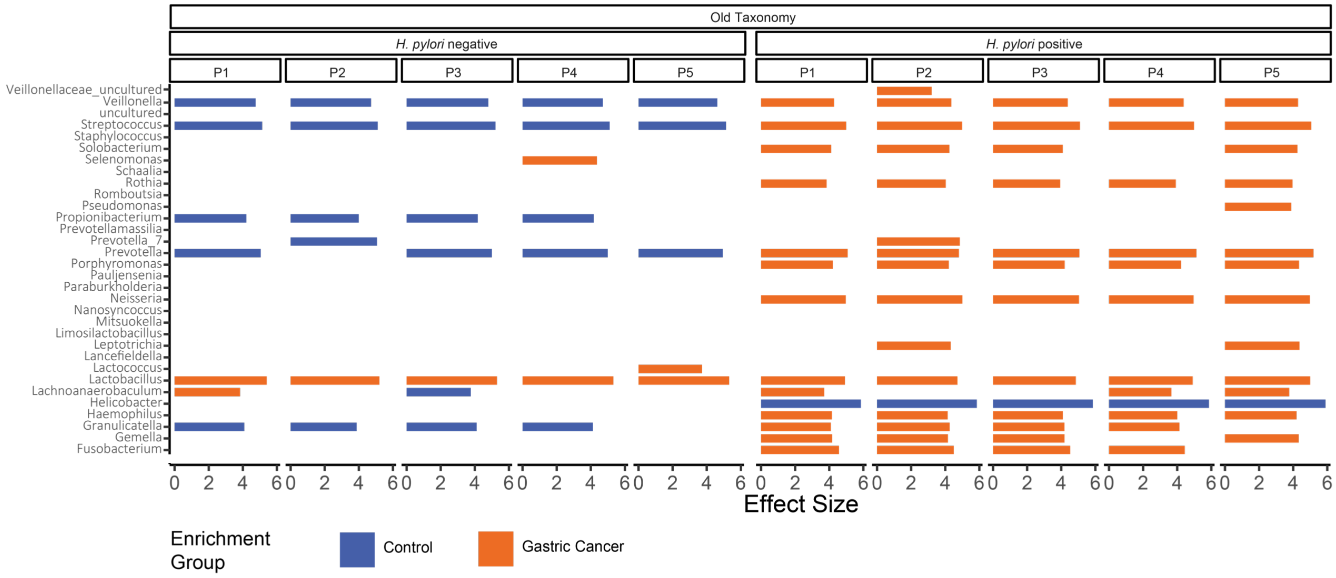

Supplementary Figure 9: Lefse analysis between control and GC sample groups based on the old taxonomic classification at genus level. All effect sizes shown reached significance level.
